# Fabrication and Characterization of Submicron-Scale Bovine Hydroxyapatite: A Top-Down Approach for a Natural Biomaterial

**DOI:** 10.3390/ma15062324

**Published:** 2022-03-21

**Authors:** Maria Apriliani Gani, Aniek Setiya Budiatin, Maria Lucia Ardhani Dwi Lestari, Fedik Abdul Rantam, Chrismawan Ardianto, Junaidi Khotib

**Affiliations:** 1 Doctoral Programme of Pharmaceutical Sciences, Faculty of Pharmacy, Universitas Airlangga, Surabaya 60115, Indonesia; maria.apriliani.gani-2019@ff.unair.ac.id; 2 Department of Pharmacy Practice, Faculty of Pharmacy, Universitas Airlangga, Surabaya 60115, Indonesia; anieksb@yahoo.co.id (A.S.B.); chrismawan-a@ff.unair.ac.id (C.A.); 3 Department of Pharmaceutical Science, Faculty of Pharmacy, Universitas Airlangga, Surabaya 60115, Indonesia; maria-lestari@ff.unair.ac.id; 4 Laboratory of Virology and Immunology, Faculty of Veterinary Medicine, Universitas Airlangga, Surabaya 60115, Indonesia; fedik-a-r@fkh.unair.ac.id

**Keywords:** submicron material, nanomaterial, bone scaffold, bone graft, calcium phosphate, neglected disease

## Abstract

Submicron hydroxyapatite has been reported to have beneficial effects in bone tissue engineering. This study aimed to fabricate submicron-scale bovine hydroxyapatite (BHA) using the high-energy dry ball milling method. Bovine cortical bone was pretreated and calcined to produce BHA powder scaled in microns. BHA was used to fabricate submicron BHA with milling treatment for 3, 6, and 9 h and was characterized by using dynamic light scattering, scanning electron microscope connected with energy dispersive X-Ray spectroscopy, Fourier-transform infrared spectroscopy, and X-ray diffractometry to obtain its particle size, calcium-to-phosphorus (Ca/P) ratio, functional chemical group, and XRD peaks and crystallinity. Results showed that the particle size of BHA had a wide distribution range, with peaks from ~5 to ~10 µm. Milling treatment for 3, 6, and 9 h successfully gradually reduced the particle size of BHA to a submicron scale. The milled BHA’s hydrodynamic size was significantly smaller compared to unmilled BHA. Milling treatment reduced the crystallinity of BHA. However, the treatment did not affect other characteristics; unmilled and milled BHA was shaped hexagonally, had carbonate and phosphate substitution groups, and the Ca/P ratio ranged from 1.48 to 1.68. In conclusion, the fabrication of submicron-scale BHA was successfully conducted using a high-energy dry ball milling method. The milling treatment did not affect the natural characteristics of BHA. Thus, the submicron-scale BHA may be potentially useful as a biomaterial for bone grafts.

## 1. Introduction

Bone defects represent a neglected form of disease that may severely interfere with the physiological function of bones [1,2,3]. This form of disease may also affect an individual’s quality of life and the patient’s economic burden [1]. Every year, more than 2.2 million people globally undergo bone grafting procedures to treat bone defects [4]. Therefore, the development of osteoconductive biomaterial should focus on bone tissue engineering to accelerate bone defect repairs.

The development of nanoscale materials has been rapidly increasing in recent decades. Nanoscale biomaterials have been reported to have beneficial effects in bone tissue engineering, such as increasing the adhesion, differentiation, and proliferation of osteoblasts and providing vascularization and bone formation in vivo [5,6]. However, the effects of nanomaterials, particularly in osteoclasts, were not as expected in osteoblasts. Chen et al. [7] reported that HA in nanoscale grain sizes (~100 nm) impaired osteoclastic formation and function. This was evidenced by inhibited cell fusion, reduced osteoclast size, increased osteoclast apoptosis, suppressed expression of osteoclast specific genes and proteins, and hampered resorption activity compared to submicron HA (~500 nm). Other than that, nanoparticles smaller than 10 nm were indicated to act similar to gas and may cross various biological barriers, reaching sensitive organs and disturbing normal cell behavior [8,9].

Furthermore, the study begins to report the beneficial effect of submicron +-scale material for bone reconstruction. The submicron +−scale material was also found to be the most influential surface dimension in promoting osteoblast differentiation, as reported by Khang et al. [10]. In addition, submicron-scale ceramic (grain size: ~800 nm) was observed to enhance bone regeneration after 12 weeks of implantation [11]. Particularly in regard to osteoclasts, it has been reported that hydroxyapatite with a particle size of ~500 nm beneficially influences osteoclast formation and function compared to smaller-sized materials [7]. Thus, considering the controversy of material sizes, it is suggested that the submicron-scale materials potentially have superior action on bone cells, particularly osteoclasts, and may be combined with other ceramics to produce desired characteristics for bone tissue regeneration [12,13,14,15].

Hydroxyapatite (HA) is a derivate of calcium phosphate that is widely used for medical purposes, including orthopedic and dental [16]. HA as a biomaterial can be synthesized from chemical precursors known as synthetic HA or extracted from natural sources such as mammalian bones [16]. One advantage in using natural HA compared to synthetic HA is the characteristics of natural HA. Natural HA, such as bovine hydroxyapatite (BHA), has a carbonate substitution group on its apatite. The natural HA is similar to human bone and was not found in synthetic HA [17,18,19]. Carbonated HA was found to increase osteoblast proliferation in vitro [20]. Other characteristics of BHA, such as its high compressive strength, prevent premature degradation and support bone formation in vivo [16,21].

Considering the beneficial effects of BHA and submicron material in bone tissue engineering, the development of submicron BHA potentially increases the osteoinductive and osteoconductive properties of BHA. As it was established that submicron material had a superior effect on osteoclasts [7], submicron BHA will also benefit bone diseases related to osteoclast dysfunction.

Studies have reported that critical parameters must be considered when fabricating hydroxyapatite [13,14,22,23]. Due to the high compressive strength property of BHA, an appropriate method should be chosen to reduce the particle size of the BHA. A previous study by Ruksudjarit et al. [24] synthesized a nano-hydroxyapatite using the wet ball milling method with ethanol as the milling media. Ethanol is widely used as a process control agent (PCA) in the milling process of biomaterials [25]. However, the use of ethanol and other organic solvents is known to be the most common source of contamination of obtained materials. These solvents reduced crystallinity, and also changed the morphology and distribution of elements of the starting materials [26,27]. Because of this, it is suggested that the use of organic compounds should be avoided in the milling treatment of biomaterials.

This study aimed to fabricate submicron-scale HA by using high-energy dry ball milling. In this study, bovine bones were given pretreatment (boiled, dried, and calcined) in order to obtain BHA. BHA naturally sized in microns was milled for several hours by using the high-energy dry ball milling method. Milled and unmilled BHA then were characterized by using dynamic light scattering (DLS, Beckman Coulter, Indianapolis, IN, USA), a scanning electron microscope connected with energy dispersive X-Ray spectroscopy (SEM-EDX, Carl Zeiss, Oberkochen, Germany), Fourier-transform infrared spectroscopy (FTIR, Bruker, Leipzig, Germany), and X-ray diffractometry (XRD, PANAlytical Corporation, Almelo, The Netherland). Submicron-scale BHA with innate characteristics of human bone has the potential to be used for bone grafts or drug delivery systems to bone tissues.

## 2. Materials and Methods

### 2.1. Extraction of Bovine Hydroxyapatite

BHA was extracted based on the methods used by Budiatin et al. [18], with modifications. The raw material was fresh cortical bone from mature bovines. Bone was cut and cleaned with water, and spongy parts and bone marrow were removed. Furthermore, the bone was boiled for 5 h in distilled water (distilled water was changed every hour). Bone was boiled in the pressurized tank for 3 h (water was changed every one hour). The boiled bone was then dried at 60 °C for three hours. The dried bone was soaked in absolute ethanol (Brataco Chemika, Surabaya, Indonesia) for 24 h while being shaken (ethanol was changed every 12 h). Calcination was conducted for two hours at 1000 °C. Finally, the bone was ground and sieved through an 80-mesh sieve.

This study also examined the BHA-based and HA-based scaffold’s compressive strength. The scaffolds were made from extracted BHA and HA (CASs number 1306-06-5, molecular weight 1004.6 g mol^−1^; further characteristics are present in Figure S1). Briefly, BHA or HA (10 g) was added to 5 mL of a prepared 20% gelatin solution (Cartino, Samut Prakan, Thailand). The mixture was stirred and sieved using a mesh (size: 1.0 mm) and dried at 37 °C. Next, 25 mg of granules were molded into an implant (diameter: 2 mm) using a hydraulic press (2 ton; Graseby-Specac Ltd., Orpington, Kent, London, UK).

### 2.2. Fabrication of Submicron-Scale BHA Using High-Energy Dry Milling

The fabrication of submicron-scale BHA was conducted based on the methods of Aminatun et al. (2019) [27]. BHA extracted from the previous method was used as the starting material. A milling ball made from alumina was used; the ratio of BHA and the milling ball was 1:20. The milling treatment was conducted for three, six, and nine hours in the milling vial.

### 2.3. Material Characterization

The compressive strength of the BHA-based and HA-based scaffolds was characterized by using a mini autograph (Autograph Microcomputer Control Universal Testing, LoadCell, YXC-1B (Original Equipment Manufacture, Surabaya, Indonesia), speed 5 mm/min).

The Ca/P ratio of materials was examined using SEM-EDX (EVO MA 10; Carl Zeiss, Oberkochen, Germany). The structural evaluation of particle size was examined using the same instrument and analyzed by measuring the mean particle size in at least two different axes using the ImageJ 1.52a software (National Institutes of Health, Bethesda, MD, USA).

The crystallinity of the materials was examined by using XRD (PANalytical X’Pert^3^ powder; PANAlytical Corporation, Almelo, The Netherland). The XRD pattern was corrected, and the XRD peaks were detected using Origin software (OriginLab Corporation, Northampton, MA, USA). The percent of crystallinity of each material was calculated based on area under peaks by using Origin software.

(1) $$\mathrm{Percent}\;\mathrm{of}\;\mathrm{crystallinity}:\;\frac{\mathrm{Acrytalline}}{\mathrm{Acrytalline}\;+\;\mathrm{Aamorphous}}\times 100\%$$

The hydrodynamic particle size of materials was measured by using DLS (Delsa™ Nano C; Beckman Coulter, Indianapolis, IN, USA), while the functional chemical group of each material was examined with FTIR (Alpha II; Bruker, Leipzig, Germany).

## 3. Results

**Figure 1:**
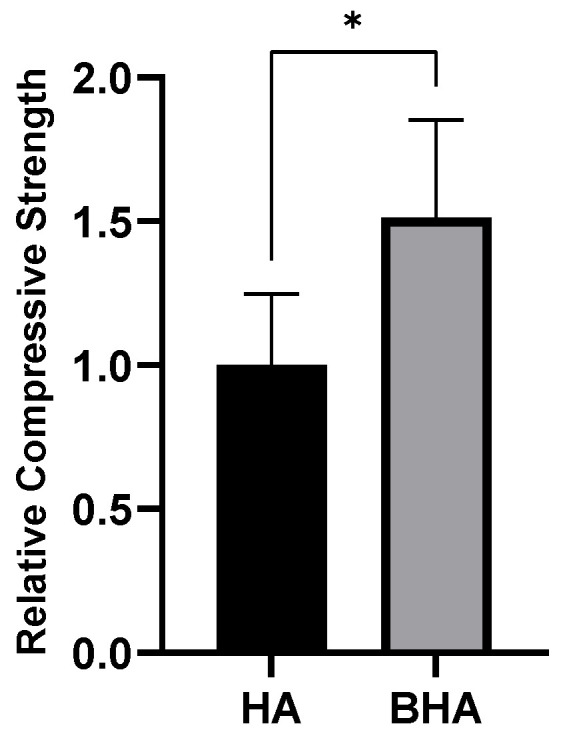
Relative compressive strength of BHA and HA. Each bar shows the mean ± *SD* ratio. * *p* < 0.05 based on the unpaired *t*-test.

Prior to the fabrication and characterization of the material, a compressive strength test was conducted to compare the strength of BHA as natural form of HA with a synthetic HA (characteristics of HA are presented in Figure S1). Figure 1 shows that BHA had a higher compressive strength than the synthetic HA (*p* = 0.0254; unpaired *t*-test). This indicated that BHA is a compact material compared to HA.

**Figure 2:**
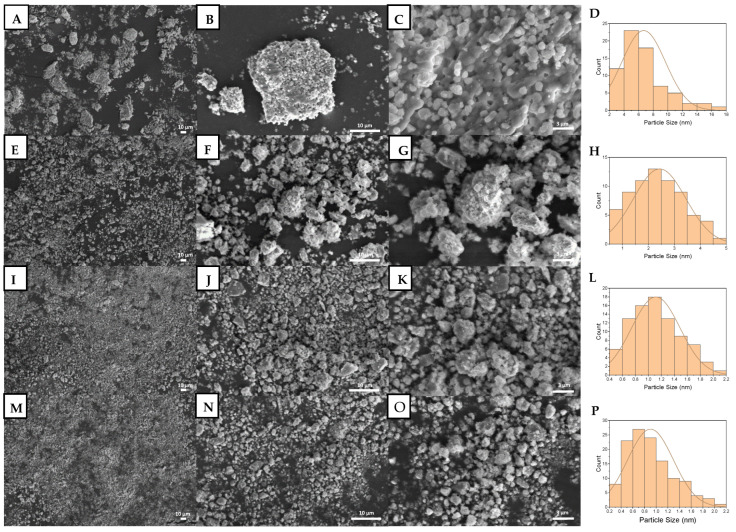
SEM images and particle size distribution of unmilled BHA (**A**–**D**), BHA milled for 3 h (**E**–**H**), BHA milled for 6 h (**I**–**L**), and BHA milled for 9 h (**M**–**P**). (**A**,**E**,**I**,**M**) Images show total magnification of 1000×. (**B**,**F**,**J**,**N**) Images show total magnification of 5000×. (**C**,**G**,**K**,**O**) Images show total magnification of 15,000×. (**D**,**H**,**L**,**P**) Graphs show the corresponding particle size distributions.

The strength of BHA indicated its brittleness, which may affect the submicron-BHA fabrication process. Thus, an appropriate method should be considered in the top-down fabrication process for BHA. In the current study, we chose the high-energy dry ball milling method to fabricate submicron BHA. Figure 2C shows that the particle size of unmilled BHA had a wide distribution range, from submicrons to ~30 µm, with a peak of ~5 to ~10 µm. Moreover, SEM images presented in Figure 2 also show that milling treatment reduced the particle size of BHA, both qualitatively and semiquantitatively. BHA samples milled for three, six, and nine hours were measured and found to have particle sizes of ~3, ~1 µm, and in the submicron scale (Figure 2F,I,L). Moreover, all material was shaped hexagonally (Figure 2); this shape of BHA also differentiates BHA from synthetic HA (Figure S1A,B).

**Figure 3:**
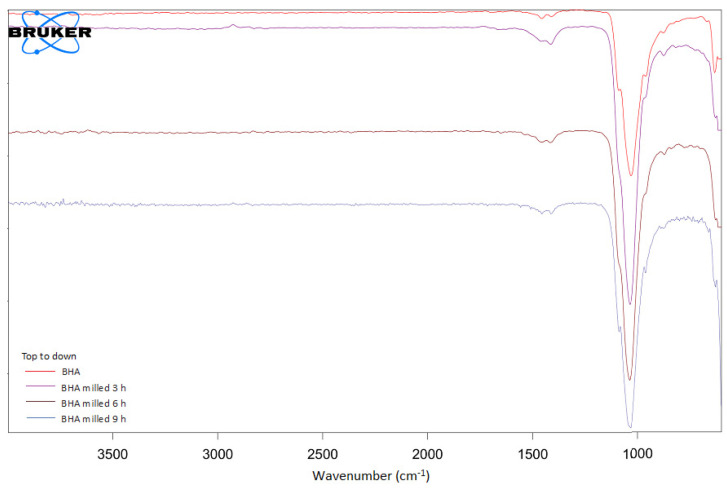
FTIR spectra of unmilled and milled BHA.

**Figure 4:**
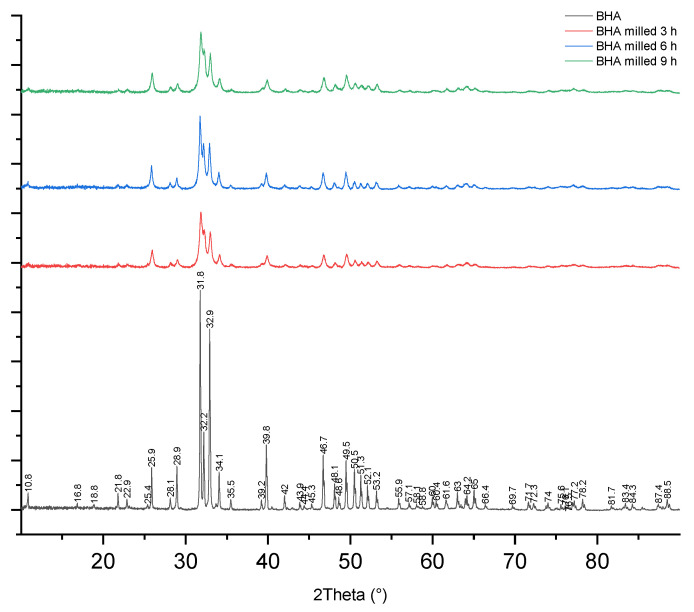
XRD spectra of unmilled and milled BHA.

Milling treatment did not affect the characteristics of BHA based on its functional chemical groups. FTIR spectra showed that all materials had carbonate and phosphate groups present at a wavenumber of 1455 cm^−1^ and 1000–1100 cm^−1^, respectively (Figure 3). Moreover, the carbonate group detected in all BHA samples was not present in HA (Figure S1C). The XRD pattern and peaks of unmilled and milled BHA are presented in Figure 4. BHA had 84.02% crystallinity, while BHA milled for 3, 6, and 9 h had 65.81%, 60.98%, and 60.70% crystallinity, respectively. In addition, the calcium-to-phosphorus (Ca/P) of unmilled and milled BHA ranged from 1.48 to 1.68 (Table 1). The Ca/P of all BHA samples (unmilled and milled) is higher than that found in synthetic HA (Figure S1E).

**Figure 5:**
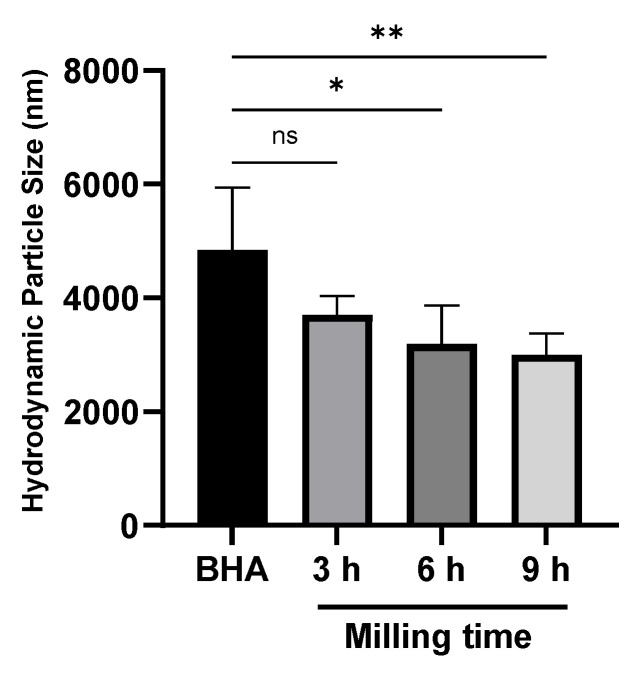
The hydrodynamic particle size of milled and unmilled BHA. Each bar shows the mean ± *SD* value. * *p* < 0.05, ** *p* < 0.01 based on a one-way ANOVA test.

The current study also conducted a hydrodynamic particle measurement of the unmilled and milled BHA (Figure 5). This measurement was essential to predict the in vivo performance of BHA as submicron materials. In line with previous results by SEM (Figure 2), the milling treatment reduced the particle size of BHA over time. However, the hydrodynamic size of milled BHA was more extensive; the particle size was scaled in micron size with BHA milled for nine hours being the smallest. 

## 4. Discussion

The current study was conducted to fabricate submicron-scale BHA using the dry ball milling method with various milling times. This milling method uses no media in the milling treatment of materials. Milling media such as ethanol and other organic solvent are widely used as PCA in the milling method [28,29,30]. PCA functions to avoid cold welding and bonding between the powder particles. However, PCA is also known to change material characteristics [25]. In the case of the milling treatment of BHA, the use of PCA may change its natural characteristics, which is not desirable. In the current study, we did not vary the milling speed because it was previously reported that milling speed did not influence the particle size of materials [31,32]. Although BHA is a material with high compressive strength, our study proved that a 9-h milling treatment successfully reduced the particle size of BHA to a submicron scale without changing the natural morphology of BHA. The hydrodynamic size of these materials was higher; however, the particle size of milled BHA was significantly smaller than unmilled BHA.

Our currently fabricated submicron BHA has been indicated to have a beneficial effect in bone tissue engineering. A previous study reported that submicron-scale materials (~500 nm) had a beneficial effect on osteoclast formation and function compared to nanomaterials [7]. This event is suggested to occur through the integrin–ligand protein interactions by protein adsorption [10]. In addition, submicron-sized ceramics (grain size: ~800 nm) have also been proven to enhance bone regeneration compared to microscale ceramics (grain size: ~2.5 µm) after 12 weeks of implantation in vivo [11].

Furthermore, our current study also proved that the milling treatment did not change the morphology of BHA. The morphology of these materials was similar to bovine-derived HA in our previous study [18] and the hydrogel composite HA in the study of Slota et al. [33]. This hexagonal-like morphology of biomaterials was reported to promote osteoblast adhesion after five hours of seeding [33]. This morphology is also present in the biomineralization of human bone mesenchymal stem cells [34], suggesting similar properties and better performance in vivo.

Moreover, our current study proved that the top-down treatment did not affect the characteristics of BHA based on its chemical functional groups. Both unmilled and milled BHA showed carbonate and phosphate chemical groups. This was similar to our previous study [18] and another study conducted by Michelot et al. [35]. The carbonate group in HA was reported to accelerate the differentiation and proliferation of osteoprogenitor cells to bone cells compared to uncarbonated HA [20]. Moreover, the carbonated HA was also reported to increase the gene expression of the collagen matrix [36]. In vivo studies also showed that carbonated HA, such as BHA, exhibited higher presentation of new bone formation and higher bone-to-material contact when compared to other types of HA [37,38].

Our current study also found that milling treatment reduced the crystallinity of BHA. Reductions in material crystallinity generally occurs because of milling treatment, such as reported by Ma et al. [39]. The study reported that a high-energy milling treatment reduced the crystallinity of MgCu. This incident is related to grain refinement and the impingement of milling balls with material, which decreased XRD peaks [39].

The Ca/P of human bone is generally considered to have a theoretical Ca/P ratio of 1.67 [40]. In the current study, we also examined the Ca/P of materials. This study showed that the Ca/P ratio of all materials was close to human HA [40]. Furthermore, the Ca/P ratio of 1.67 is also widely used as a reference value for bone grafts, rendering it suitable for orthopedic, dental, and maxillofacial implants [41,42]. Thus, the Ca/Pa value of fabricated submicron BHA supported the potential of this material to be used for bone implants. Moreover, calcium and phosphate play essential roles in bone tissue formation. Calcium ions stimulate the osteoblastic bone synthesis pathway, affects osteoblasts’ life span, and regulate the formation and the resorptive functions of osteoclasts. On the other hand, phosphate regulates the differentiation and growth of osteoblastic lineage, increases the expression of bone morphogenetic proteins, and plays a role in the maturation of osteocytes [43,44].

To the best of our knowledge, our current study is the first study that considers the milling treatment of hard and dense natural HA without PCA. The non-hazardous method of milling treatment used in this study reduced the natural particle size of the material without affecting its characteristics. Considering the beneficial effects of submicron material and the characteristics of natural HA, submicron-sized natural HA may have beneficial effects in bone tissue engineering.

## 5. Conclusions

Submicron-scale natural HA fabrication was successfully conducted using a high-energy dry ball milling method. A milling time of 9 h decreased the particle size of BHA from micron to submicron scale. The milling treatment did not affect the natural characteristics of BHA marked by the morphology, chemical group substitution, crystallinity, and Ca/P ratio of the material. Thus, the submicron-scale BHA with innate characteristics of human bone may be potentially used as a biomaterial that could possibly have better in vitro and in vivo performance. However, further research regarding this should be proven in further studies.

## Figures and Tables

**Table 1:** Ca/P ratio of unmilled and milled BHA.

Material	Calcium (Ca)		Phosphorus (P)		Ca/P Ratio
	Weight (%)	Atomic (%)	Weight (%)	Atomic (%)	
BHA	68.01	62.16	31.99	37.84	1.64
BHA milled 3 h	68.43	62.62	31.57	37.38	1.68
BHA milled 6 h	65.70	59.69	34.30	40.21	1.48
BHA milled 9 h	65.95	59.95	34.05	40.05	1.50

## Data Availability

Not applicable.

## Supplementary Materials

The following supporting information can be downloaded at https://www.mdpi.com/article/10.3390/ma15062324/s1, Figure S1: Characteristics of synthetic HA.
