# Supplementary material for: Fabrication and Characterization of Submicron-Scale Bovine Hydroxyapatite: A Top-Down Approach for a Natural Biomaterial
Source: Materials (Basel). 2022 Mar 21;15(6):2324. doi: 10.3390/ma15062324 (PMC8953508; doi:10.3390/ma15062324)
Supplement: Supplementary file 1 [file materials-15-02324-s001.zip › materials-1609555-supplementary.pdf]

Supplementary

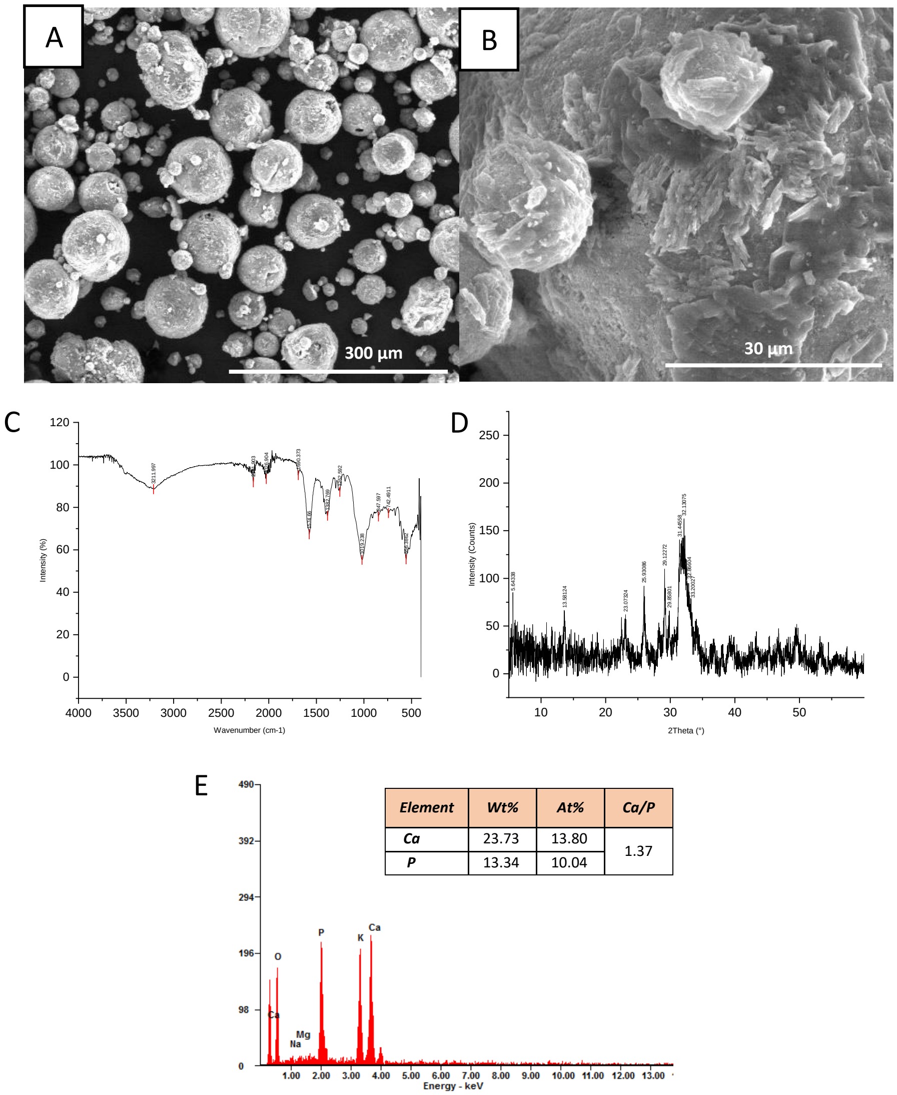

Figure S1. Characteristics of synthetic HA. SEM images (A-B), FTIR spectra (C), XRD spectra (D), elemental composition from EDX.
